# Viral DNA genomes in sera of farrowing sows with or without stillbirths

**DOI:** 10.1371/journal.pone.0230714

**Published:** 2020-03-26

**Authors:** Caroline Tochetto, Ana Paula Muterle Varela, Diane Alves de Lima, Márcia Regina Loiko, Camila Mengue Scheffer, Willian Pinto Paim, Cristine Cerva, Candice Schmidt, Samuel Paulo Cibulski, Lucía Cano Ortiz, Sidia Maria Callegari Jacques, Ana Cláudia Franco, Fabiana Quoos Mayer, Paulo Michel Roehe

**Affiliations:** 1 Laboratório de Virologia, Departamento de Microbiologia, Imunologia e Parasitologia, Instituto de Ciências Básicas da Saúde, Universidade Federal do Rio Grande do Sul, Porto Alegre, Rio Grande do Sul, Brazil; 2 Laboratório de Virologia, Faculdade de Veterinária, Universidade Federal do Rio Grande do Sul, Porto Alegre, Rio Grande do Sul, Brazil; 3 Laboratório de Biologia Molecular, Centro de Pesquisa em Saúde Animal, Instituto de Pesquisas Veterinárias Desiderio Finamor, Secretaria Estadual de Agricultura, Pecuária e Desenvolvimento Rural, Eldorado do Sul, Rio Grande do Sul, Brazil; 4 Departamento de Estatística, Instituto de Matemática e Estatística, Universidade Federal do Rio Grande do Sul, Porto Alegre, Rio Grande do Sul, Brazil; Plum Island Animal Disease Center, UNITED STATES

## Abstract

A study was conducted to investigate the serum virome of sows with and without stillbirths after farrowing. Sera from sows with at least one stillbirth or with normal litters were collected immediately after farrowing. Viral DNA was extracted from serum pools and submitted to high throughput sequencing. No differences in the proportion of virus-related reads were found in both groups (p > 0.05). A variety of viral DNA genomes were identified, mostly representative of three viral families: *Anelloviridae*, *Circoviridae* and *Smacoviridae*. Besides, a number of novel unclassified circular Rep-encoding single stranded DNA (CRESS DNA) viruses were also identified. These findings suggest that the presence of such viral genomes in sows’ sera bears no correlation with stillbirths’ occurrence; it seems likely that these constitute part of the normal serum microbiome of sows at farrowing.

## Introduction

In swine reproduction, the term “stillbirth” refers to piglets with healthy appearance born dead at term. The average incidence of stillbirths in commercial farms may range between 3% to 8%, causing significant losses to swine producers [1–3]. Such condition is considered a multifactorial problem [4]; on occasions, stillbirths have been associated to a number of known pathogens, including bacteria, protozoa, and viruses. Among viruses, those which have been most often associated with stillbirths are porcine parvovirus (ungulate protoparvovirus 1, UPV1) [5], porcine circovirus type 2 (PCV2) [6], Aujeszky's disease virus (ADV), Influenza virus [7], porcine reproductive and respiratory syndrome virus (PRRSV) [8], encephalomyocarditis virus (EMCV) [9] and classical swine fever virus (CSFV) [10]. Nevertheless, frequently, no viruses or other known pathogens seem to play a role in such condition [1, 2, 11, 12].

To date, most studies focusing on viruses with potential association with stillbirths have used traditional approaches, such as serological tests on samples from sows or stillborn piglets. More recently, the polymerase chain reaction (PCR) has been employed in this kind of studies [1, 11]; however, this method is still unable to detect agents to which no previously published genome sequences are available. Currently, high throughput sequencing (HTS) has been applied to investigate the full content of nucleic acids in a sample with no need for previous knowledge of specific nucleic acid sequences. As such, HTS has become a suitable tool to investigate the participation of infectious agents, in the etiology of conditions such as stillbirths. In this study, an investigation was conducted in search for DNA viruses in the serum of sows. Serum samples were collected in commercial swine farms in Southern Brazil from sows soon after farrowing and submitted to high throughput sequencing. Associations between the detection of DNA viral genomes and the occurrence of stillbirths were investigated. This work led to the detection of a number of complete viral genomes, which are here reported.

## Material and methods

### Ethics statement

This study was approved by the Ethics Committee in the Use of Animals from the Veterinary Research Institute *Desidério Finamor* (CEUA-IPVDF)–protocol number 16/2015.

### Sampling and study design

Just farrowed sows from six commercial piglet producing farms located in five municipalities in Southern Brazil were selected. The sows were derived from farms certified as free of classical swine fever, pseudorabies, brucellosis, tuberculosis and mange. All sows were vaccinated to porcine circovirus 2, porcine parvovirus (i.e. ungulate protoparvovirus), *Haemophilus parasuis*, *Erysipelothrix rhusiopathiae*, colibacilosis and leptospirosis. All animals were clinically healthy at the time of sampling. Blood samples were taken from the auricular vein using disposable syringes and transported to the laboratory on ice. Sera were separated from blood and processed according to previously described protocols [13]. Sows from each farm were divided in two groups. The first included sows which had at least one stillbirth at this farrowing (named “S”; n = 6); the second consisted of sows with no stillbirths (named “H”; n = 6). Each group consisted of a pool with different numbers of serum samples. On total, serum samples of 94 sows were collected and grouped in twelve pools (six “S” and six “H”) for sequencing (S1 Table). Sows which had abortions or mummified fetuses at farrowing were not included in this study.

### Sample processing and sequencing

After centrifugation (~ 5,000 x g/5 min), serum samples were pooled and filtered through 0.45 and 0.22 μm filters (Millex-GV, PVDF, Millex^®^). These were then centrifuged on a sucrose cushion (25%) at an average 150,000 x g for 4 h at 4 °C. The obtained pellets were resuspended in 400 μL of milli-Q water and stored at -80 °C until further processing. Subsequently, the resuspended pellets were treated with DNase (2 U/μL, Turbo DNase Kit, Ambion) and RNase A (20 mg/mL, Invitrogen) to reduce the concentration of non-encapsidated nucleic acids [14]. The samples were then incubated for 2 h at 37 °C and each half (200 uL) used for either DNA or RNA extraction. Viral DNA was extracted using a standard phenol protocol [15]. The DNA was enriched by multiple displacement amplification (MDA) using ϕ29 DNA polymerase [16]. Viral RNA was extracted with TRIzol (Invitrogen, Carlsbad, CA) according to the manufacturer’s protocol and enriched by whole transcriptome amplification (WTA) using REPLI-g^®^ WTA Single Cell Kit (Qiagen). Both preparations were then purified with AMPure XP magnetic beads (Agentcourt). The quality and quantity of the enriched nucleic acids was checked by microvolume spectrophotometry (Nanodrop, ThermoScientific, USA) and fluorimetry (Qubit, Invitrogen, USA), respectively. The quantity of enriched RNA obtained after purification was insufficient for HTS; therefore, only DNA was sequenced. Twelve libraries (one for each pool) were constructed using the Nextera DNA preparation kit (Illumina^®^) according to the manufacturers’ recommendations. The sequencing was performed using the Miseq v2 300 kit (2 x 150 paired-end).

### Bioinformatics pipeline

The quality of the obtained reads was verified using FastQC software (http://www.bioinformatics.babraham.ac.uk/projects/fastqc/). Low quality reads (phred quality score <20) were trimmed using PRINSEQ tool (v. 0.20.4) [17]. The trimmed reads were *de novo* assembled using metaSPAdes assembler (v. 3.9.0) with default settings [18]. Assembled contigs were compared to the viral protein database of GenBank using BLASTx (*e-value* cutoff of 10^−5^) with Blast2GO software. Sequences with the best BLAST score were selected and assigned into known viral families and when applicable, to the respective CRESS DNA viruses current groups. Viral hits classified as bacteriophages were further submitted to PHASTER [19] to confirm their identities. Annotation and visualization of circular genomes were performed in Geneious software (v.8.1.3). Genome coverage was obtained by mapping raw reads using low-sensitivity/fastest mode (Geneious, v.8.1.3).

### Assessment of viral abundance

The number of raw reads matching selected viral sequences (contigs and concatenates previously classified by BLAST) were measured using the Geneious assembly tool, with all raw data output reads in the “low-sensitivity/fastest” mode. Virus-specific reads from each pooled sample were then normalized by the total number of viral reads generated in each pool. Differences between the data obtained from pools of sows with and without stillbirths were compared using the Wilcoxon T test. A *p-value* <0.05 was indicative of statistical significance. A Principal Component Analysis (PCA) was performed to compare the virome profile between the groups. All such analyses were carried out with the SPSS software version 22 (IBM, Armonk, NY, USA).

### Phylogenetic analyses

Phylogenetic analyses were performed based on predicted amino acid sequences (for circular rep-encoding ssDNA viruses, CRESS DNA) or nucleotide sequences (for *Torque teno sus virus*–TTSuV). Multiple sequence alignments were performed with the MEGA7 software [20] using ClustalW [21] with default settings to CRESS DNA sequences and with modifications for TTSuV (gap open creation penalty of 10 and gap extension penalty of 5). A phylogenetic tree of TTSuV was inferred from p-distance matrix using neighbor-joining method with a bootstrap of 1000 interactions. For CRESS DNA, the phylogenetic tree was inferred by maximum likelihood method using the best fit substitution model (VT + G + I + F) determined by PhyML through AIC criterion with approximate likelihood branch support (aLRT) [22].

### Nucleotide sequence accession numbers

The viral genomes described here were deposited in GenBank under accession numbers MH170056 to MH170073.

## Results and discussion

### Virome analysis

The twelve pools generated a total of 7,918,254 reads (S2 Table). The overall percentage of eukaryotic viral reads was 29.50% in sows with stillbirths and 29.91% in sows without stillbirths. Reads corresponding to bacteriophages represented 0.03% of obtained reads and were identified in only four pools (1S, 3S, 4S, 6H). One of the challenges of virome analysis by HTS is to obtain enough amounts of viral nucleic acid free of host or bacterial DNA contamination. This was overcome here, since the rates of viral reads were higher than previously reported in swine serum [23] and faeces [24].

**Fig 1:**
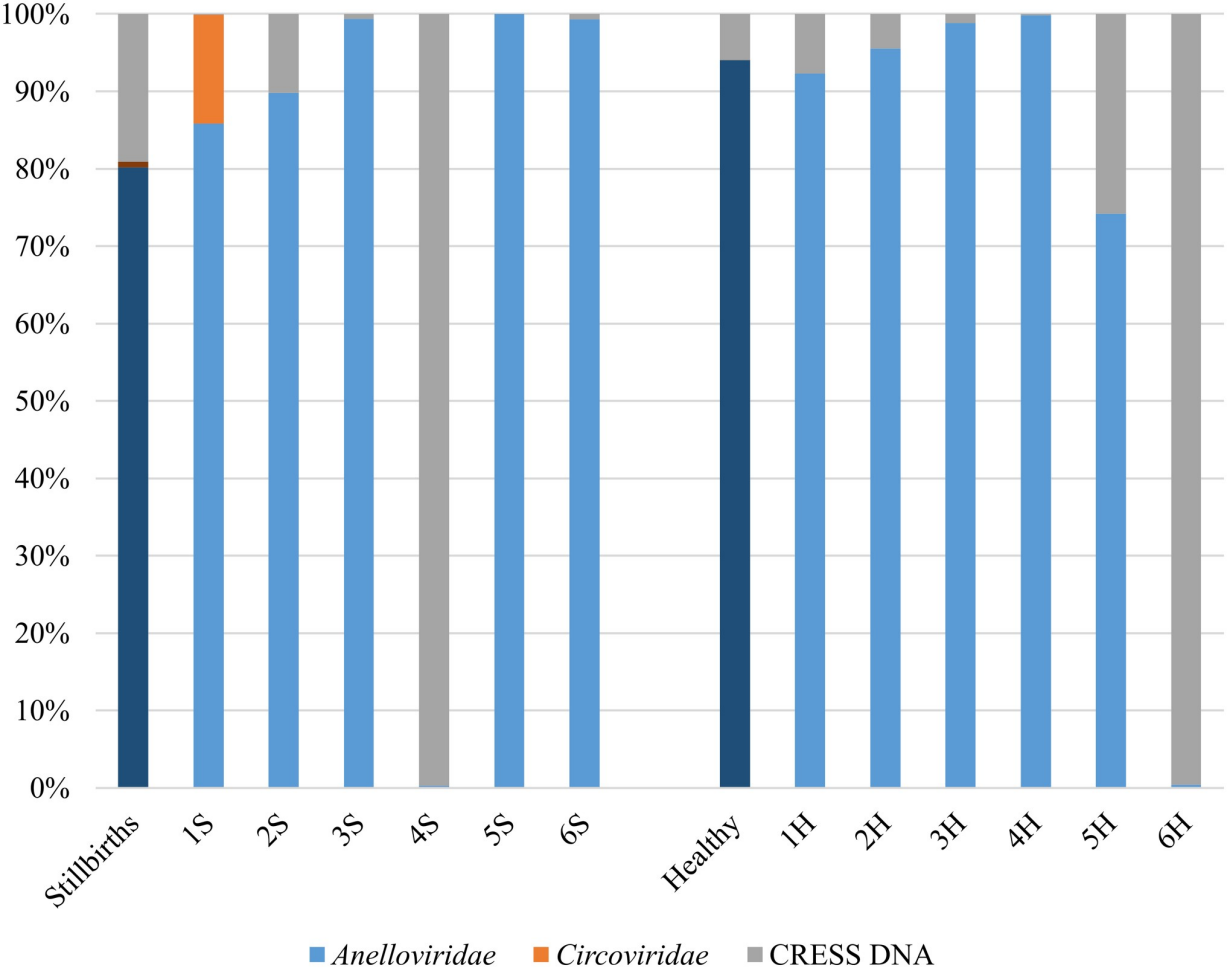
Distribution of sequence reads matching different eukaryotic viral families in each group. The number of reads corresponding to each family was normalized by the number of eukaryotic viral reads. Dark blue columns represent the average reads obtained in each of the groups. The numbers followed by a letter in parenthesis correspond to the identification of the pools analyzed in this study. Numbers correspond to each of the farms (1–6); letters S (stillbirth) or H (healthy) refer with the occurrence of stillbirths. Reads related of *Circoviridae* were identified in three pools: 1S, 3S and 3H.

Reads related to two eukaryotic viral families with DNA genomes (*Anelloviridae* and *Circoviridae*), plus a number of circular Rep-encoding single stranded DNA (CRESS DNA) viruses were identified (Fig 1). The most abundant reads were related to torque teno sus viruses (TTSuV), members of *Anelloviridae* family, which were detected in all examined pools. Although many studies have attempted to link TTSuVs to disease in swine, their role in pathological conditions, if any, is still under debate, as TTSuVs have been detected circulating in healthy and diseased pigs at high viral loads [25–27]. Regarding reproductive failure, fetuses infected with TTSuV have been already identified; however, no associations between TTSuV and the occurrence of abortion or other reproductive problems have, to date, been proven [28]. Therefore, the participation of such viruses in reproductive disease seems unlikely.

The second most frequently detected reads were those of CRESS DNA viruses (Fig 1), which were detected in eleven out of the twelve serum pools. Currently, the International Committee on Taxonomy of Viruses (**ICTV**) classified CRESS DNA viruses associated with eukaryotic hosts into four families: *Circoviridae*, *Genomoviridae*, *Geminiviridae*, *Nanoviridae*, *Bacilladnaviridae* and *Smacoviridae* [29]. These have been detected in a wide variety of samples, including faeces of pigs from many countries [30–36]; however, this is the first description of CRESS DNA viruses in sows’ sera. It seems likely that such viruses constitute part of the normal swine microbiota. What would be the role for CRESS DNA viruses circulating in the blood of pregnant sows is a query that remain unanswered.

The third most frequently detected reads were representatives of the *Circoviridae* family (*Porcine circovirus type 1* —PCV1—and *Porcine circovirus type 3*—PCV3). These were detected in three pools (1S, 3S, 3H), although in pools 3S and 3H these represent less than 1% of the reads (Fig 1). PCV1 reads were identified in low proportions in two pools: one of sows with stillbirths (3S) and other of sows without stillbirths (3H). On the other hand, PCV3 reads were identified in two pools from sows with stillbirths (1S and 3S). Nevertheless, no association could be drawn between detection of circovirus genomes and stillbirths. Subsequent studies revealed that PCV3 genomes were highly prevalent in sera of most sows sampled here and showed no association with the occurrence of stillbirths (Tochetto et al., 2019 *submitted*).

**Fig 2:**
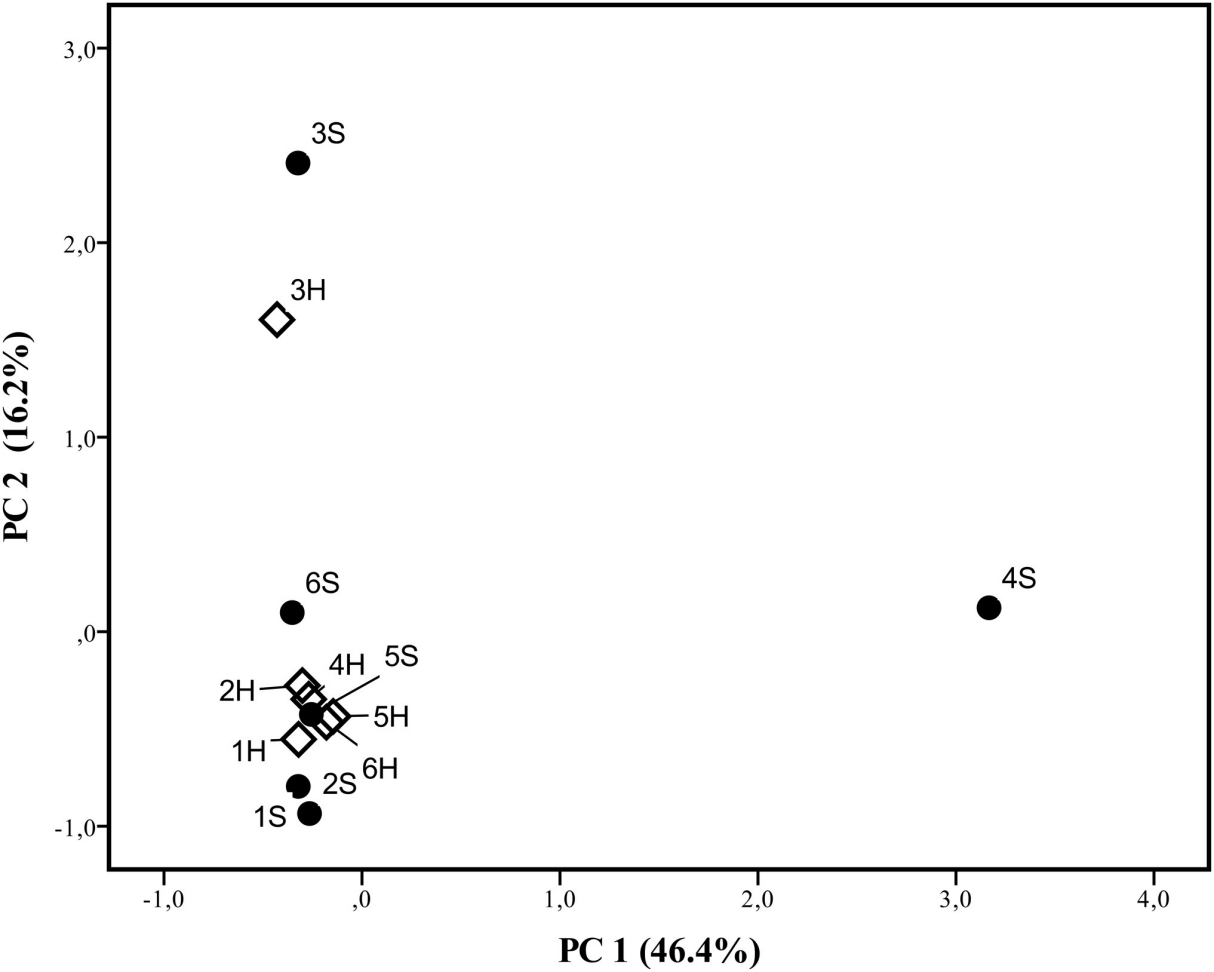
Principal Component Analysis (PCA) score plots of virus-specific reads on sera from sows with and without stillbirths. The first two principal components (PC1 and PC2) were maintained since they explained more than 62% of the observed variability. Black dots represent the pools of sera from sows with stillbirths (S); white diamonds represent the pools of sera from sows without stillbirths (H).

When comparing the viral serum profile on both groups (with and without stillbirths), no statistically significant differences (p > 0.05) were observed (Table 1). Likewise, no distinct patterns could be detected by PCA (Fig 2). These findings support the lack of association between serum viromes and the occurrence of stillbirths. It is important to note that due to the large diversity of virus observed, a “core” virome could not be identified in the samples, which in part may have hampered the identification of specific viral patterns according to the group. Nevertheless, some viruses were detected exclusively in the group of sows without stillbirths (porcine stool-associated circular virus, hudisavirus, PCV3 and unclassified CRESS DNA viruses). However, due to the low amount of reads recovered, no statistically significant differences were achieved between the presence of such genomes in sows with or without stillbirths (Table 1).

**Table 1:** Wilcoxon T test for non-parametric related data on viral reads detected in serum pools from sows with (S) and without (H) stillbirths.

Virus	Stillbirths		Healthy		p-value
	Median	(Min—Max)	Median	(Min—Max)	
*Anelloviridae*	9,46E-01	(2,50E-03–1,00E+00)	9,40E-01	(3,20E-03–9,98E-01)	0.92
TTSuV 1b	0,00E+00	(0,00E+00–2,24E-01)	0,00E+00	(0,00E+00–9,00E-04)	0.65
TTSuV 1a	8,70E-01	(2,50E-03–1,00E+00)	9,40E-01	(3,20E-03–9,98E-01)	0.75
*Circoviridae*	0,00E+00	(0,00E+00–1,39E-01)	0,00E+00	(0,00E+00–1,00E-04)	0.18
Porcine circovirus 3	0,00E+00	(0,00E+00–1,39E-01)	0,00E+00	(0,00E+00–0,00E+00)	0.18
Porcine circovirus 1	0,00E+00	(0,00E+00–2,00E-05)	0,00E+00	(0,00E+00–1,00E-04)	0.32
CRESS DNA	7,00E-03	(0,00E+00–9,96E-01)	6,10E-02	(1,80E-03–8,89E-01)	0.60
Rat stool associated circular virus	0,00E+00	(0,00E+00–2,93E-02)	4,00E-03	(0,00E+00–4,87E-02)	0.50
Porcine stool associated circular virus	0,00E+00	(0,00E+00–9,91E-02)	0,00E+00	(0,00E+00–0,00E+00)	0.18
Porcine serum associated circular virus	1,50E-03	(0,00E+00–3,85E-02)	6,00E-04	(0,00E+00–2,30E-03)	0.14
Po-circo-like virus	5,00E-04	(0,00E+00–2,88E-01)	3,00E-02	(0,00E+00–8,89E-01)	0.17
Odonata associated circular virus	0,00E+00	(0,00E+00–3,60E-01)	0,00E+00	(0,00E+00–2,00E-03)	0.65
Hudisavirus	0,00E+00	(0,00E+00–1,52E-02)	0,00E+00	(0,00E+00–0,00E+00)	0.32
Duck faeces associated circular virus	5,00E-04	(0,00E+00–8,92E-02)	2,00E-04	(0,00E+00–5,80E-03)	0.69
Dromedary stool associated circular virus	0,00E+00	(0,00E+00–1,80E-03)	0,00E+00	(0,00E+00–1,80E-03)	0.32
Unclassified CRESS DNA viruses	0,00E+00	(0,00E+00–1,95E-01)	0,00E+00	(0,00E+00–0,00E+00)	0.18

No statistically significant difference was detected between the groups (p>0.05); the number of viral reads were divided by the total number of reads for each pool.

The viruses which are often associated with stillbirths, i.e. UPV1, PCV2, PRRVS, EMCV, CSFV and Influenza virus, were not detected in this study. It must be pointed that the sows were vaccinated to UPV1 and PCV2; therefore, genomes corresponding to these agents were not expected to be detected. Viral RNA genomes were not obtained since they could not be recovered from the samples. This might have been caused either by the true absence of viral RNA genomes in samples, or by its presence in such small quantities that it could have been missed by the extraction procedure. However, the protocol employed here is well stablished in the laboratory and has demonstrated efficiency at viral RNA extraction [37,38]. Considering the good health status of the sows during sampling, it is possible that RNA viruses were not circulating in the animals. Whereas PRRSV is exotic to the country [39], EMCV causes a disease of rare occurrence in swine [40] and CSFV has been eradicated in the sampled area [41]. Sows from which samples were collected were from certified CSFV-free farms. Therefore, these agents were not expected to be detected in such samples.

It must should be considered that HTS does not provide an actual quantification of the detected viromes. Biases such as those introduced by the enrichment method (MDA) must be born in mind, although the impact of MDA on HTS has been lessened and regarded as not significant enough to lead to data misinterpretation [42]. Circular ssDNA genomes are preferentially amplified by MDA, and this could have interfered with the apparent abundance of such viral genomes detected in the present study However, such abundance certainly contributed to provide additional credibility to the six new CRESS DNA genomes discovered here.

DNA viruses which are often associated with stillbirths, as UPV1 and PCV2, were not detected in this study. It must be pointed that the sows were vaccinated to UPV1 and PCV2; therefore, genomes corresponding to these agents were not expected to be detected.

It is important to highlight that, in the present study, the samples were examined in pools, which may hamper the detection of genomes present in low concentrations. It is possible that sequencing individual samples might provide some additional information. In addition, only viral genomes matching the GenBank virus database were evaluated here. Most of the reads (~ 72%) could not be related to previously published viral sequences; these may represent new genomes of viruses, as yet unknown, infectious agents. Such sequences should be subject of further investigation.

The findings reported here reveal a number of viral genomes circulating in the serum of sows at farrowing. These strongly suggest that there is no association between the presence of such viral genomes in sow’s sera at farrowing and the occurrence of stillbirths. Nevertheless, it is still possible that an as yet undetected virus (or other infectious agents), could putatively be associated to stillbirths, though just not present in sow’s sera at farrowing. Viremia is expected to precede transplacental infection [43]. However, infection might have occurred throughout the gestation period and not be detected in sows’ sera at farrowing.

### Complete viral genomes

Altogether, a total of 20 complete viral genomes were recovered from sows either with or without stillbirths, including twelve TTSuV, six CRESS DNA viruses and two PCV3 genomes. PCV3 genomes were recovered from two different pools of sows with stillbirths (farms 1 and 3) and were described elsewhere [13]. However, no association could be detected between PCV3 infection and the occurrence of stillbirths.

Next, a brief report is provided on the full viral genomes detected in this study.

#### Anelloviruses

**Fig 3:**
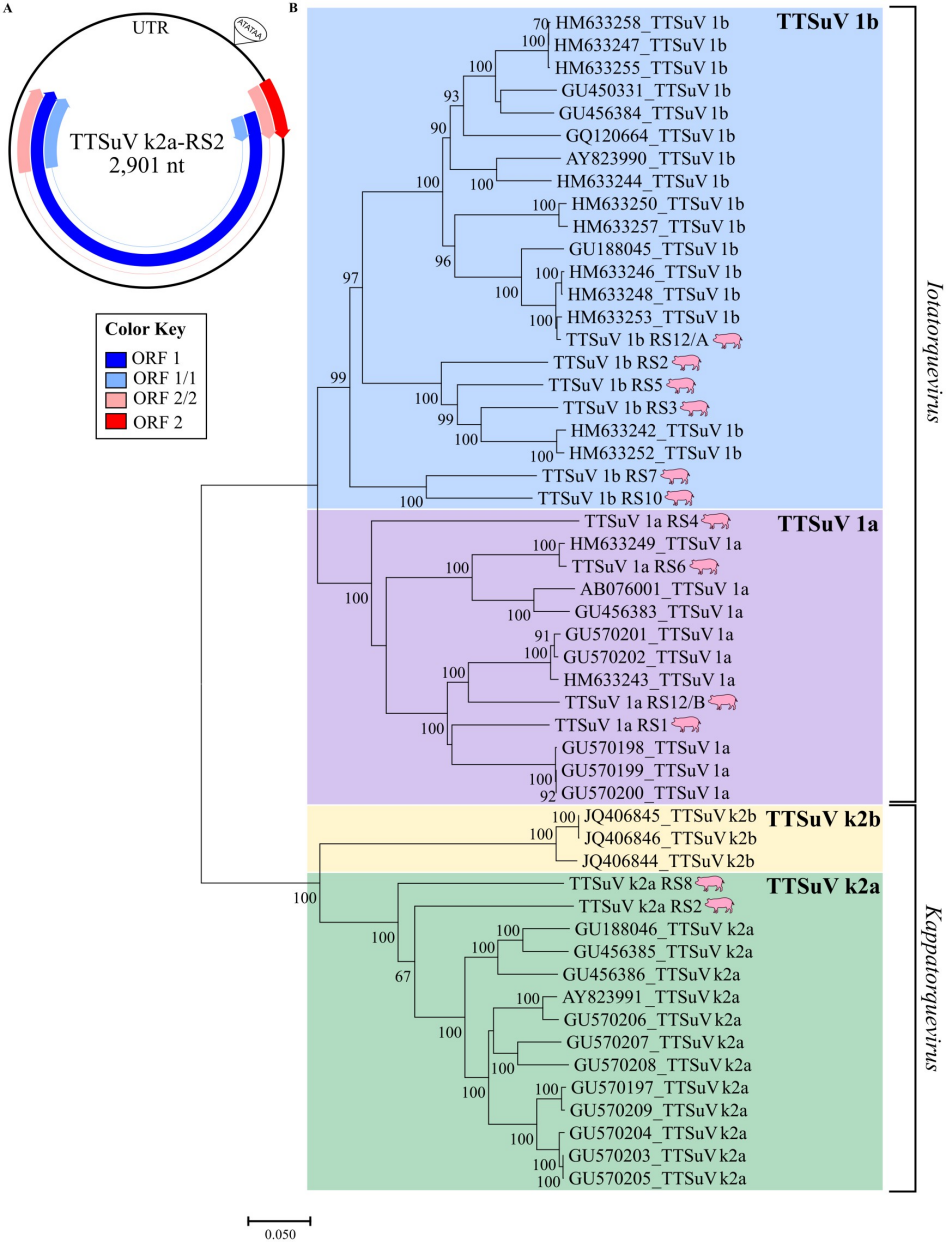
The TTSuV genomes recovered in this study. (A) Schematic representation of one out of the twelve TTSuV genome recovered in this study. (B) Phylogenetic analysis based on the entire ORF1 at nucleotide level. Neighbor-joining method with p-distance and a bootstrap of 1000 replicates. Drawings of pigs highlight the sequences recovered in this study.

All twelve genomes representative of members of the *Anelloviridae* corresponded to TTSuV: four TTSuV 1a, six TTSuV 1b and two TTSuV k2a. The three TTSuV species were identified in sera of sows with (S) and without (H) stillbirths (Table 2). The TTSuV genome sizes varied between 2.8 to 2.9 kb. Four open reading frames (ORF) were identified: ORF1, ORF2, ORF1/1 and ORF2/2 (Fig 3A). ORF 1/1 and ORF 2/2 share the same intron of ORF1 and ORF2, respectively. The UTR length varied between 706–819 nt among TTSuV 1a, 696–824 nt among TTSuV 1b, and 880–939 nt between TTSuV k2a. Typical domains in the UTR region as the TATA box (motif ATATAA) and the G-C rich region were also detected.

**Table 2:** Genomes of TTSuV recovered in this study.

Sequence name	Genus	Accession number	Length	^a^Mean coverage	^b^Sample
TTSuV 1a RS/1	*Iotatorquevirus*	MH170062	2,910	7444.3	1H
TTSuV 1b RS/2	*Iotatorquevirus*	MH170063	2,930	7305.2	2H
TTSuV 1b RS/3	*Iotatorquevirus*	MH170064	2,904	3761.8	3H
TTSuV 1a RS/4	*Iotatorquevirus*	MH170065	2,803	2198.6	4H
TTSuV 1b RS/5	*Iotatorquevirus*	MH170066	2,906	1388.2	5H
TTSuV 1a RS/6	*Iotatorquevirus*	MH170067	2,882	3326.6	1S
TTSuV 1b RS/7	*Iotatorquevirus*	MH170068	2,891	1399.0	2S
TTSuV 1b RS/10	*Iotatorquevirus*	MH170069	2,887	1207.3	5S
TTSuV 1b RS/12/A	*Iotatorquevirus*	MH170070	2,868	6558.7	6S
TTSuV 1a RS/12/B	*Iotatorquevirus*	MH170071	2,910	6455.1	6S
TTSuV k2a RS/2	*Kappatorquevirus*	MH170072	2,901	15.3	2H
TTSuV k2a RS/8	*Kappatorquevirus*	MH170073	2,826	2270.6	3S

^a^Genome coverage = average of nucleotides/site; obtained by mapping raw reads to reference.

^b^Farms sampled are numbered (1 to 6). Letters refer to groups with (S) or without (H) stillbirths; e.g.; 1S = farm 1, sows with stillbirths; 1H = farm 1, sows without stillbirths.

Based on the criteria for TTSuV classification from ICTV (2015), multiple alignment analyses of the entire ORF1 were performed. Phylogenetic analyses were performed based on the entire ORF1. The twelve TTSuV ORF1 sequences reported here were compared to 40 previously available TTSuV sequences. In such phylogenetic reconstruction, six of the ORF1 sequences identified here clustered along with TTSuV 1b; four clustered with TTSuV 1a, whereas two clustered along with TTSuV k2a (Fig 3B).

The TTSuV recovered here share 36.6% to 84.7% nucleotide identity, revealing two distinct genera. The TTSuV 1a sequences share 64.1–82.5% nucleotide identity, whereas the TTSuV 1b sequences shared 65.1–84.7% nucleotide identity in relation to each other. The two TTSuV k2a sequences exhibited a nucleotide sequence identity of 71.5%.

#### CRESS DNA genomes

Six complete CRESS DNA genomes were recovered from four different serum pools, collected in three of the farms (Table 3). Five of the full CRESS DNA genomes identified do not fall within any family previously reported CRESS DNA viruses; thus, these were named “porcine serum associated circular DNA viruses” (PoSCV-1 to -5). One genome representative of a member of a recently created family of CRESS DNA genomes, named *Smacoviridae* [29], genus *Porprismacovirus* (as in “porcine and primate smacovirus”), was identified and named Porcine associated porprismacovirus 3, 12/RS/BR (Table 3).

**Table 3:** Genomes of CRESS DNA viruses recovered in this study and best amino acid hit sequence of each identified ORF.

Accession number	Acronym	^b^Pool	^c^Genome coverage	ORF	^d^Best hit	Identity	E-value	Cover
MH170056	^a^PoSCV-1 2A RS/BR	2H	10.5	Rep	Duck faeces associated circular DNA virus 2 (NC_030133)	86%	0.0	99%
				Cap	Porcine serum-associated circular virus (KU203356)	53%	1E-160	100%
MH170057	PoSCV-2 2B RS/BR	2H	47	Rep	Odonata-associated circular virus -17 (KM598400)	63%	2E-108	92%
				Cap	Odonata-associated circular virus -17 (KM598400)	34%	2E-15	75%
MH170058	PoSCV-3 7A RS/BR	2S	123.7	Rep	Duck faeces associated circular DNA virus 2 (NC_030133)	86%	0.0	96%
				Cap	Duck faeces associated circular DNA virus 3 (NC_030134)	67%	1E-180	100%
MH170059	PoSCV-4 7B RS/BR	2S	27.3	Rep	Hudisavirus sp. (MF351519)	89%	0.0	98%
				Cap	Hudisavirus sp. (MG522858)	78%	1E-158	87%
MH170060	PoSCV-5 8 RS/BR	3S	142.9	Rep	Porcine serum-associated circular virus (KU203352)	94%	0.0	100%
				Cap	Porcine stool-associated circular virus 7 (KJ577814)	57%	2E-137	100%
MH170061	Porcine associated porprismacovirus 3, 12/RS/BR	6S	37.3	Rep	Porcine stool-associated circular virus 2 (KJ577818)	85%	0.0	100%
				Cap	Porcine stool-associated circular virus 3 (LC133374)	93%	0.0	100%

^a^PoSCV = Porcine serum associated circular DNA virus.

^b^The numbers refer to the farms; letters (“S” for stillbirths; “H” for healthy) refer to group; e.g.; 2S = farm 2, sows with stillbirths; 2H = farm 2, sows without stillbirths.

^c^Genome coverage = average of nucleotides/site; obtained by mapping raw reads to reference.

^d^Based on BLASTp search.

**Fig 4:**
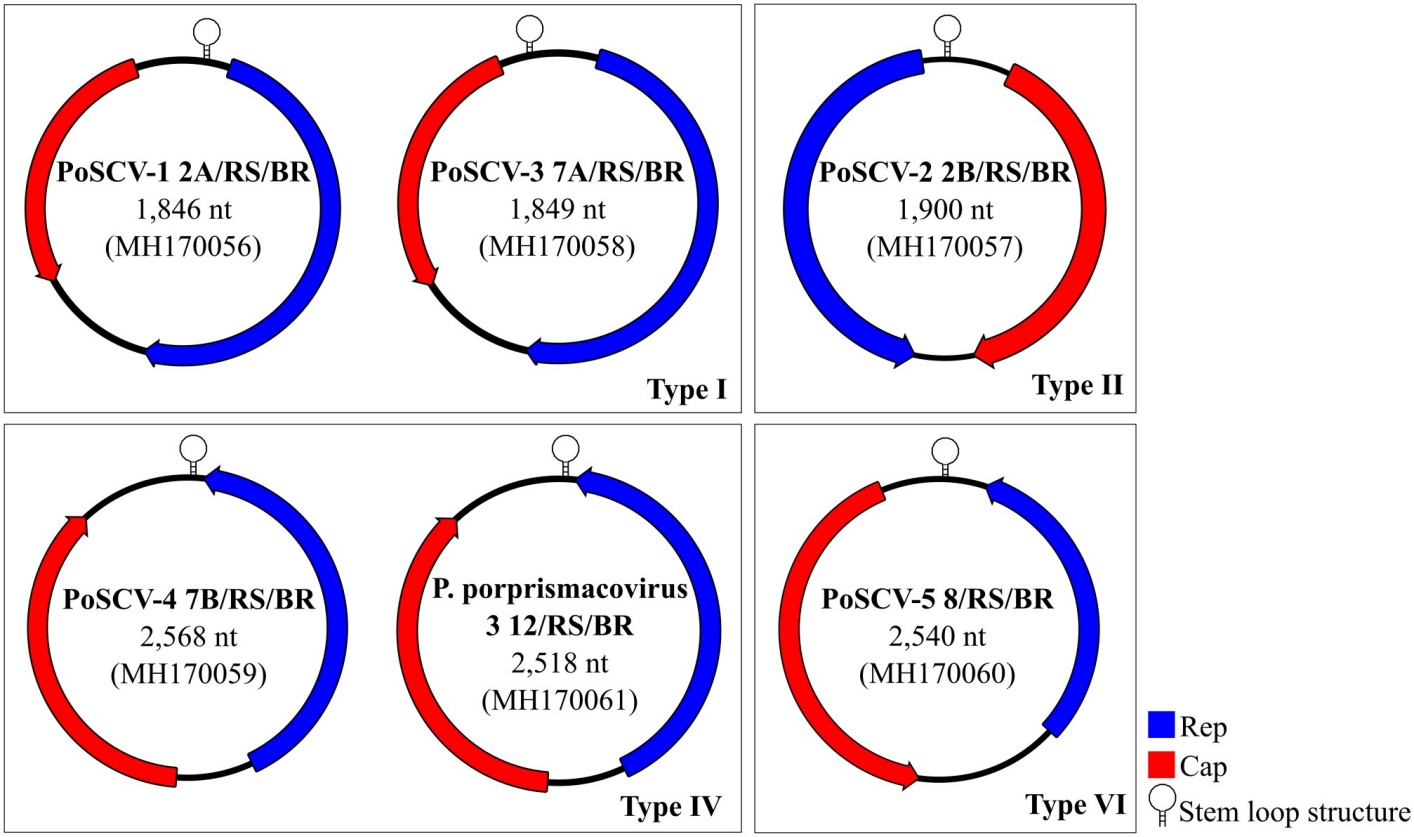
The genomic organization of the six CRESS DNA genomes recovered from serum of sows with (S) and without stillbirths (H).

**Fig 5:**
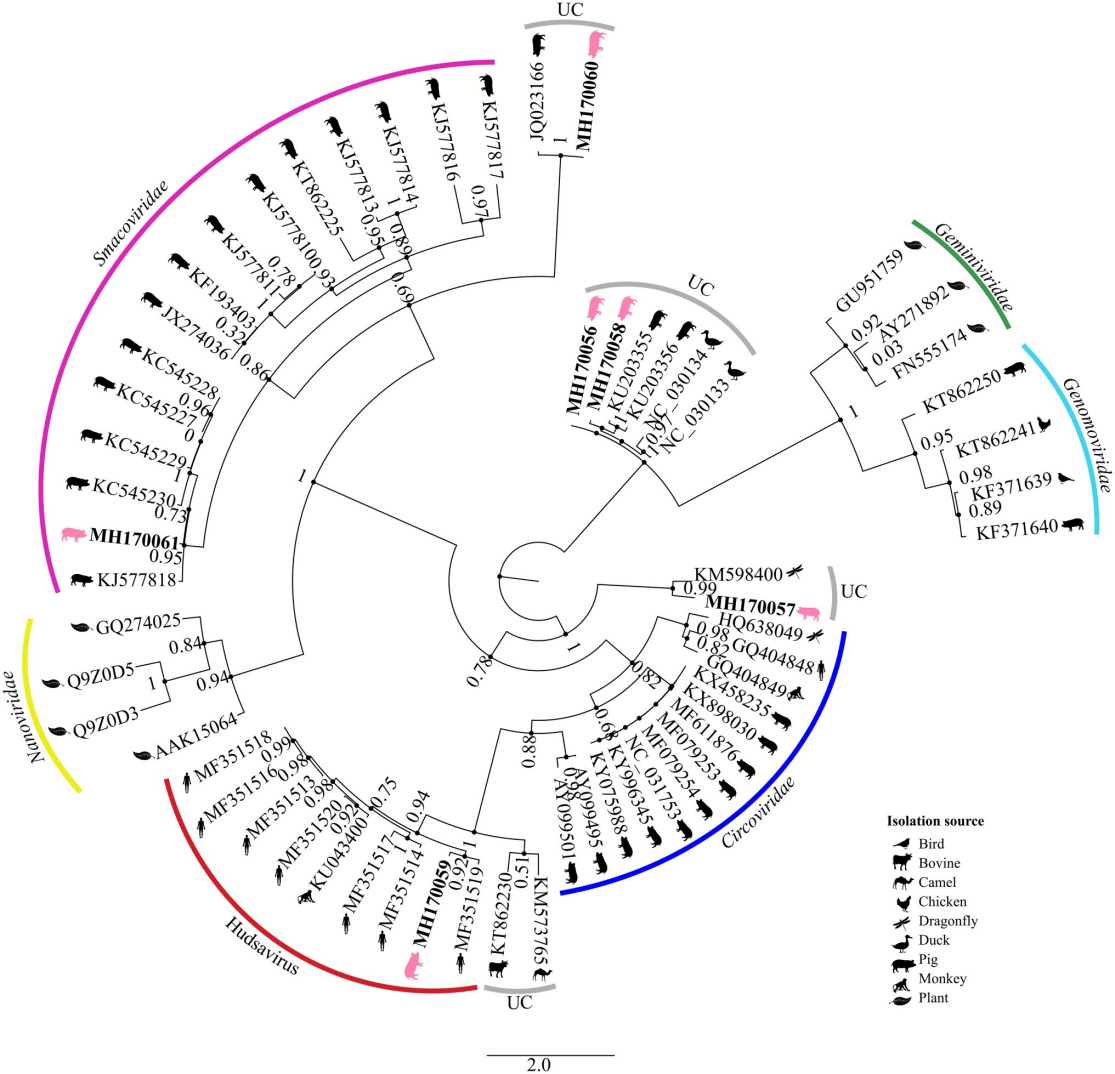
Maximum-likelihood phylogenetic tree of Rep amino acid sequences of CRESS DNA viruses. Phylogenetic tree was inferred using PhyML with the VT + G + I + F substitution model. Sequences recovered in this study are highlighted by pig drawings. The number represents the farm and the letter the group; e.g. 2S = farm 2, sows with stillbirths; 2H = farm 2, sows without stillbirths.

The CRESS DNA genomes recovered here ranged in length from 1,846 to 2,568 nt, with two major ORFs that encode the putative Rep and Cap proteins. Four types of genome architectures previously described [44] were identified: type I (n = 2), type II (n = 1), type IV (n = 2) and type VI (n = 1) (Fig 4). All genomes revealed a putative origin of replication (*ori*) that contains a nonanucleotide motif at the apex of a predicted stem-loop structure responsible for initiating the rolling-cycle replication [44]. A phylogenetic tree was reconstructed based on Rep amino acid sequences reported here, with the best matches of BLASTp in GenBank and those of representative CRESS DNA genomes, including members of *Smacoviridae*, *Genomoviridae*, *Gemiviridae*, *Circoviridae* and *Nanoviridae* (Fig 5). PoSCV-1 2A/RS/BR, PoSCV-2 2B/RS/BR, PoSCV-3 7A/RS/BR and PoSCV-5 8/RS/BR clustered within different groups of unclassified CRESS DNA. *Porcine associated porprismacovirus 3* 12/RS/BR (MH170061) clustered with members of *Smacoviridae* family, and PoSCV-4 7B/RS/BR clustered with sequences of Hudisavirus (Fig 5).

PoSCV-2 2B/RS/BR and PoSCV-4 7B/RS/BR exhibited a conserved nonanucleotide motif (NANTATTAC), while PoSCV-1 2A/RS/BR, PoSCV-3 7A/RS/BR and PoSCV-5 8/RS/BR displayed less common nonamers (Table 4). Rolling circle replication (RCR) motifs I, II, and III and superfamily 3 helicase (SF3) Walker A, B, and C motifs were also identified in all of them, except for PoSCV-2 2B/RS/BR and PoSCV-5 8/RS/BR, which lack the motif I (Table 4).

**Table 4:** Conserved motifs identified in the Rep putative amino acid sequence of CRESS DNA viruses recovered in this study.

Genome	Nanonucleotide motif	RCR motifs			SF3 helicase motifs		
		I	II	III	Walker-A	Walker-B	Motif C
PoSCV-1 2A/RS/BR	TAATATTAA	LLTYPQ	EHYHA	YVKK	GKTGTGKT	IFDDI	IFTS
PoSCV-2 2B/RS/BR	TAGTATTAC	-	KHIHI	YIEK	GDSGKGKT	VIEEF	YTCN
PoSCV-3 7A/RS/BR	AAATATTAA	LLTYPQ	EHYHA	YVKK	GATGTGKT	IFDDI	IFTS
PoSCV-4 7B/RS/BR	CAGTATTAC	CFTINN	PHYQG	YCRK	APPGTGKS	VFEEF	ITSN
PoSCV-5 8/RS/BR	AGCAAGCAA	-	RHYQF	YVYK	EKGNSGKT	IIDTP	ILCN
Porcine associated porprismacovirus 3 12/RS/BR	TAGTATTAC	MATIPH	KHIQC	YEKK	SEGNSGKT	IIIDI	CMTN

PoSCV-1 2A/RS/BR and PoSCV-3 7A/RS/BR were most closely to sequences of CRESS DNA detected in serum of Brazilian pigs (KU203355, KU203356) and in duck feces in New Zealand (NC030133, NC030134) (Fig 5). They shared both the RCR and SF3 helicase motifs described in the genome of faeces of ducks, named associated circular DNA virus 2 (DufCV-2), except for one variation in the second aa in the Walker-A motif in PoSCV 3 7A/RS/BR (Table 4).

The PoSCV-2 2B/RS/BR genome showed no significant nucleotide identity to any of the previously known CRESS DNA genomes available on GenBank database. Despite this, PoSCV-2 genome contains characteristics identified in others CRESS DNA viruses. This genome of 1,900 nt in length is bidirectionally organized, encoding a putative Cap on the virion-sense strand and a putative Rep on the complementary strand. The large intergenic region (181 nt) contains a predicted stem-loop located at the 5’ end of Rep, with a conserved nonanucleotide at the apex (TAGTATTAC) (Fig 4). The Rep protein have RCR motifs II and III and the SF3 helicase domains Walker A, Walker B and motif C (Table 4). The absence of the RCR motif I had already been reported in CRESS DNA viruses [32]. The function of the RCR motif I is not fully understood, but it is believed to be important in DNA binding and cleavage prior to RCR [32, 44, 45]. Additionally, the Rep of PoSCV-2 shares 63% amino acid identity with Odonata-associated circular virus-17 (KM598400), identified in dragonflies in USA.

Another unclassified CRESS DNA genome, named PoSCV-4 7B/RS/BR shares ~78% nucleotide identity with hudisavirus (MF351515). These viruses were recently discovered in samples of human diarrheal feces in Peruvian patients [46], but their pathogenic potential remains unclear. Similar to hudisavirus, PoSCV-4 contains an ambisense genome that encodes a putative Cap on the virion-sense and a Rep on the complementary sense (Fig 4). Indeed, PoSCV-4 exhibited similar characteristics with hudisavirus, such as the nanomer (NANNNTTAC) at the apex of a stem-loop, the RCR and SF3 helicase motifs (Table 4). Very similar RCR motifs were also described in an unclassified CRESS DNA recovered from bovine faeces (BofCV-1) in New Zealand (KT822230). The SF3 helicase motifs of PoSCV-4 were the same of MF351515 and BofCV-1, except for one change in the first amino acid of the Walker-A motif (G/A). Finally, PoSCV-5 8/RS/BR was most closely related to Porcine serum-associated circular virus (KU203352) identified in Brazilian pig sera, with which the Rep coding region shares 94% identity (coverage 100%; e-value 0.0).

#### Smacoviruses

The recently proposed family of CRESS DNA viruses, *Smacoviridae*, comprises genomes that contain two major ORFs organized in opposite directions, encoding the rolling circle replication-associated protein (Rep) on the complementary strand and the capsid protein (Cap) on the virion-sense strand. Recently, smacoviruses were divided into six new genera, including one that represents smacoviruses recovered from pigs and primates, named *Porprismacovirus* (**Por**cine and **pri**mate **smacovirus**) [29]. Out of the six full CRESS DNA genomes identified here, one genome shares more than 82% genome-wide pairwise identity with *Porcine associated porprismacovirus 3* (KC545227-30). Following the criteria for classification of smacovirus, that stablishes a threshold of 77% genome-wide pairwise identity for species demarcation, this genome was named Porcine **associated** porprismacovirus 3 12/RS/BR. The RCR and SF3 helicase motifs detected in this genome were the same identified in previously reported genomes representatives of the same species (Table 4). Smacovirus genomes recovered from pigs were reported in USA, South Korea and New Zealand [31,33–35]; however this is the first report of such virus genome in serum of pigs.

## Conclusions

This study provides a picture of DNA viruses present in sera of just farrowed sows with or without cases of stillbirths, in which genomes of anelloviruses, circoviruses and CRESS DNA viruses were the most abundantly detected. No association could be established between any of the viral genomes here identified and the occurrence of stillbirths. Nevertheless, a number of novel viral genomes were identified; these seem to be part of the normal virome in the serum of farrowing sows.

## Supporting information

S1 Table Identification of serum pools. Farm and city of origin of sows, occurrence/non-occurrence of stillbirths, and numbers of sows sampled in each pool are shown.
(DOCX)

S2 Table Number of reads obtained by high throughput sequencing. Viral eukaryotic reads and bacteriophage reads were normalized by the number of viral reads in each pool.
(DOCX)
