# Supplementary material for: Viral DNA genomes in sera of farrowing sows with or without stillbirths
Source: PLoS One. 2020 Mar 26;15(3):e0230714. doi: 10.1371/journal.pone.0230714 (PMC7098587; doi:10.1371/journal.pone.0230714)
Supplement: S1 Table — Farm and city of origin of sows, occurrence/non-occurrence of stillbirths, and numbers of sows sampled in each pool are shown. (DOCX) [file pone.0230714.s001.docx]

**S1 Table – Identification of serum pools**. Including farm and city of origin of sows, occurrence/non-occurrence of stillbirths, and numbers of sows sampled in each pool.

| **Farm** | **City** | **Pool identification** | |  | **Number of sampled sows** | | **Total** |
| --- | --- | --- | --- | --- | --- | --- | --- |
|  |  | With stillbirths (S) | Without stillbirths (H) |  | S | H |  |
| 1 | Salvador das Missões | 1S | 1H |  | 8 | 5 | **13** |
| 2 | Encantado | 2S | 2H |  | 5 | 8 | **13** |
| 3 | São Pedro do Butiá | 3S | 3H |  | 6 | 10 | **16** |
| 4 | Cruzeiro do Sul | 4S | 4H |  | 6 | 8 | **14** |
| 5 | São Pedro do Butiá | 5S | 5H |  | 3 | 12 | **15** |
| 6 | Serafina Corrêa | 6S | 6H |  | 11 | 12 | **23** |
|  | **Total** | **12 pools** | |  | **39** | **55** | **94** |
