# Supplementary material for: Viral DNA genomes in sera of farrowing sows with or without stillbirths
Source: PLoS One. 2020 Mar 26;15(3):e0230714. doi: 10.1371/journal.pone.0230714 (PMC7098587; doi:10.1371/journal.pone.0230714)
Supplement: S2 Table — Viral eukaryotic reads and bacteriophage reads were normalized by the number of viral reads in each pool. (DOCX) [file pone.0230714.s002.docx]

**S2 Table – Number of reads obtained by high throughput sequencing.** Viral eukaryotic reads and bacteriophage reads were normalized by the number of viral reads in each pool.

| **Group** | **Farm** | **Raw Reads** | **Trimmed reads** | **Viral reads (%)** | **Viral eukaryotic viral reads** | **Phage reads (%)** | **Viral contigs** |
| --- | --- | --- | --- | --- | --- | --- | --- |
| Stillbirths | 1S | 357,306 | 354,870 | 75,292 (21.07) | 74,510 (98.96) | 782 (1.04) | 480 |
|  | 2S | 356,708 | 353,796 | 30,334 (8.50) | 30,334 (100) | 0 (0) | 53 |
|  | 3S | 459,648 | 455,804 | 448,369 (97.55) | 448,071 (99.93) | 298 (0.07) | 73 |
|  | 4S | 2,109,788 | 2,049,608 | 252,705 (11.98) | 252,407 (99.88) | 298 (0.12) | 243 |
|  | 5S | 753,334 | 721,992 | 32,040 (4.25) | 32,040 (100) | 0 (0) | 48 |
|  | 6S | 613,632 | 599,286 | 534,449 (87.10) | 534,449 (100) | 0 (0) | 189 |
| Healthy | 1H | 550,300 | 549,020 | 188,912 (34.33) | 188,912 (100) | 0 (0) | 156 |
|  | 2H | 709,508 | 692,062 | 370,864 (52.27) | 370,846 (100) | 0 (0) | 240 |
|  | 3H | 459,154 | 453,738 | 108,332 (23.59) | 108,329 (100) | 0 (0) | 107 |
|  | 4H | 437,636 | 429,362 | 102,587 (23.44) | 102,587 (100) | 0 (0) | 138 |
|  | 5H | 567,848 | 562,406 | 37,173 (6.55) | 37,173 (100) | 0 (0) | 111 |
|  | 6H | 543,392 | 537,688 | 7,090 (1.30) | 6,323 (89.18) | 767 (10.82) | 50 |
| **Total** |  | **7,918,254** | **7,759,632** | **2,188,147** | **2,185,981** | **2,166** | **1,888** |
